# Dysphagia in cervical spinal cord injury: How international literature trends can guide South African practice patterns – A scoping review

**DOI:** 10.4102/sajp.v77i1.1542

**Published:** 2021-05-10

**Authors:** Kim A. Coutts

**Affiliations:** 1 Department of Speech Pathology, Faculty of Humanities, University of the Witwatersrand, Johannesburg, South Africa

**Keywords:** dysphagia, spinal cord injury, speech pathology, practice patterns, multidisciplinary, interdisciplinary, scoping review, scope of practice

## Abstract

**Background:**

The limited data regarding dysphagia in high-level spinal cord injuries (SCIs) stem from economically developed countries. Dysphagia is prevalent in patients with cervical SCI; however, in a South African context, speech-language pathologists (SLPs) are not seen as key when managing this population. This may result in patients not being screened or identified early, leading to possible complications. The literature could provide useful insight on how best to address this clinical gap.

**Objectives:**

The aim of my study was to conduct a scoping review on the description of dysphagia, the risk factors for developing dysphagia post-SCI and the practice patterns of team members working with dysphagia in cervical SCIs.

**Methods:**

A five-step scoping review was undertaken. Data were analysed by using descriptive statistics as well as a thematic analysis by using a top-down approach.

**Results:**

Through the process of screening according to the inclusion and exclusion criteria, 25 articles were included. Primarily, the pharyngeal phase was affected, which can lead to an aspiration pneumonia. The key risk factors were the presence of a tracheostomy tube, the use of ventilation and anterior spinal cord surgery. There was little mention regarding specific practice patterns, but an interdisciplinary approach was suggested as the most efficient model.

**Conclusions:**

Specific guidelines and management options need to be considered for a South African context, given the high incidence of trauma-related injuries. There needs to be locally produced research, providing suggestions on how different team members can screen and identify dysphagia within this population. Solutions need to be unique, and contextually responsive and appropriate.

**Clinical implications:**

The team members and the roles of these different team members need to be re-examined in order to ensure the early identification and management of cervical SCI patients who are at risk of developing a dysphagia.

## Background

Dysphagia (swallowing disorder) is heterogeneous in nature because of the complexity of the normal swallow physiology (Cichero 2006). The human swallow requires the use of 25 pairs of muscles as well as cortical and sub-cortical involvement (Cichero 2006). From this neurological control stems the fine temporal coordination of the breath–swallow cycle that ensures the adequate closure of the airway to prevent aspiration (Nishino 2012). Prevention of aspiration is vital in the cervical spinal cord injury (SCI) population, which presents with impaired respiratory function. This is because people with a high SCI present with impaired respiration and are therefore unable to adequately clear their airway (Wolf & Meiners 2003). A person who then presents with a resultant pneumonia with already impaired respiratory function would be subject to a serious medical complication that can result in a variety of sequalae such as but not limited to increased hospital stay, pneumothorax or even death (Lynch et al. 2017).

There is little epidemiological data relating to SCIs in South Africa (SA), despite trauma-related injuries being considered as one of the quadruple burdens of disease (Pillay-Van Wyk et al. 2016), but it is reported that around 75 million people have experienced some forms of SCIs (Joseph et al. 2017). Literature trends stemming from a variety of economically developed countries, such as the United States of America and others in Europe, indicate that dysphagia is prevalent in the SCI population, but prevalence rates vary from 26% to 80% (Papadopoulou et al. 2013; Valenzano, Waito & Steele 2016). One of the primary difficulties that occurs as a result of a higher (above C5) SCI is that of respiratory complications (Hagen 2015) including: insufficiency of respiratory muscle functioning, resulting in long-term ventilation and possible tracheostomy tube insertion; reduced vital capacity; and ineffective cough, pneumothorax and pleural effusion (Hagen 2015). These respiratory complications potentially can lead to the inability of patients to adequately protect their airway during the swallow as their normal respiratory cycle has been compromised (Martin-Harris et al. 2005). As these patients are also more prone to developing pneumonia, the prevention of a further respiratory compromise from an aspiration should be prioritised and requires the input from multiple healthcare professionals.

In a resource-restricted country such as SA, the early identification of dysphagia needs to be highlighted to reduce the medical comorbidities that can arise from an aspiration and subsequently reduce the patient’s length of stay in often-under-resourced healthcare facilities. In SA, speech-language pathologists (SLPs), who manage dysphagia, are not typically the primary members involved in the management of cervical SCIs. Given the complexities of the South African healthcare system as well as the suggested prevalence and heterogeneity of dysphagia within this population, the early identification and management require a unique team approach, which is currently lacking. Furthermore, when considering the imminent implementation of the National Health Insurance (NHI), there will also be a need to develop new care packages for patients with an SCI. By using published trends as a guide, South African healthcare professionals can start to develop these specific management options that are contextually appropriate and responsive. Thus, the objective of this scoping review was to describe the presentation of dysphagia in cervical patients with an SCI, the risk factors and resultant sequalae associated with it and then to describe the healthcare team models that have been used in the management thereof.

## Method

Scoping reviews are not intended to assess the quality of the studies as does a systematic review (Levac, Colquhoun & O’Brien 2010) and require a less rigid protocol compared with a stringent systematic review because of the intended outcomes that are different (Thomas et al. 2017).

The scoping review methodology as set out by Levac et al. (2010) was used for my study after the five stages of data collection and analysis. As this was a scoping review, no ethical clearance was required.

**FIGURE 1:**
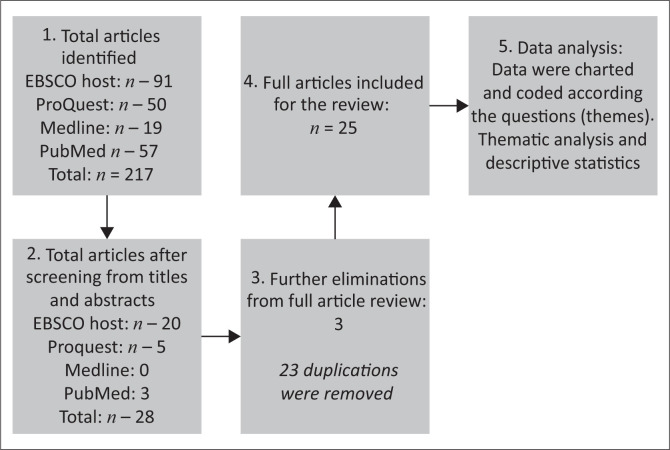
Data collection process.

MeSH terms as suggested by Baumann (2016) were utilised in EBSCOhost, ProQuest, Medline and PubMed to conduct the search. The following terms with truncation were used: *dysphagia *deglutition disorder *spinal cord injury *tetraplegia. This search yielded a total of 217 studies from all databases. Studies included, needed to be published within the last 15 years in peer-reviewed journals and were required to be in English. Opinion papers, unpublished theses or grey literature were not included. Duplications were removed and the abstracts of the remaining studies were then screened for applicability. After further eliminations from the full article screening, 25 articles were reviewed for analysis. Data were analysed by using a descriptive analytic method that comprised simple descriptive statistics and a thematic content analysis. Braun and Clarks’ (2006) data analysis methodology was used for the thematic analysis (Braun & Clark 2006) by using a top-down approach as themes were already identified according to the objectives of my study. Collating, summarising and reporting of the results were performed according to the objectives of my study. The data collection process can be seen in Figure 1. The 25 articles that were included in the review are described in Table 1 with a brief description of their demographics.

**TABLE 1:** Articles included in the scoping review.

Author	Year	Title	Study type
Valenzano et al.	2016	A review of dysphagia presentation and intervention following traumatic spinal cord injury: An understudied population	Systematic review
Iruthayarajah et al.	2018	Risk factors for dysphagia after a spinal cord injury: A systematic review and meta-analysis	Systematic review
Papadopoulou et al.	2013	Dysphagia associated with cervical spinal and postural disorders	Systematic review
Shem et al.	2012	Dysphagia and respiratory care in individuals with tetraplegia: Incidence, associated factors and preventable complications	Prospective study
McRae et al.	2019	Oropharyngeal dysphagia management in cervical spinal cord injury patients: An exploratory survey of variations due to care across specialised and non-specialised units	Survey
Shem et al.	2011	Dysphagia in individuals with tetraplegia: Incidence and risk factors	Prospective study
Ihalainen et al.	2017a	Risk factors for laryngeal penetration-aspiration in patients with acute traumatic cervical spinal cord injury	Prospective study
Lee et al.	2016	A bitter pill to swallow: Dysphagia in cervical spinal cord injury	Retrospective review
Ihalainen et al.	2017b	Traumatic cervical spinal cord injury: A prospective clinical study of laryngeal penetration and aspiration	Prospective study
Shem et al.	2019	Pharyngeal dysphagia in individuals with cervical spinal cord injury: A prospective observational cohort study	Prospective study
Hayashi et al.	2020	The time course of dysphagia following traumatic cervical spinal cord injury: A prospective cohort study	Prospective study
Sedl et al.	2010	Dysphagia in acute tetraplegics: A retrospective study	Retrospective study
Pattison, Kincaid & Pandaya	2017	Cervical fractures: Does injury level impact the incidence of dysphagia in elderly patients?	Record review
Hayashi et al.	2017	Risk factors for severe dysphagia in acute spinal cord injury	Prospective study
Brady et al.	2004	Predictors to dysphagia and recovery after cervical spinal cord injury during acute rehabilitation	Prospective study
Kirschblum et al.	1999	Predictors of dysphagia after spinal cord injury	Prospective study
Wolf and Meiners	2003	Dysphagia in patients with acute cervical spinal cord injury	Cross sectional study
Shin et al.	2011	Dysphagia in cervical spinal cord injury	Retrospective study
Chaw et al.	2012	Dysphagia and associated respiratory considerations in cervical spinal cord injury	Prospective study
Abel, Ruf and Spahn	2004	Cervical spinal cord injury and deglutition disorders	Prospective study
Ryu et al.	2012	Evaluation of dysphagia after cervical spinal cord surgery using laryngeal electromyography	Prospective study
Rihn et al.	2011	What is the incidence and severity of dysphagia after anterior cervical surgery?	Prospective study
Bailey et al.	2016	Severe dysphagia requiring gastrostomy following cervical spine fracture fixation	Retrospective study
Martin, Neary and Diamant	1997	Dysphagia following anterior cervical spine surgery	Prospective study
Vining et al.	2017	Interdisciplinary rehabilitation for a patient with incomplete cervical spinal cord injury and multimorbidity	Prospective study
Shem et al.	2012	Diagnostic accuracy of bedside swallow evaluation versus videofluroscopy to assess dysphagia in individuals with tetraplegia	Prospective study

The majority of the articles, 11 (44%), were published within the last 5 years, followed by 10 (40%) within the last 10 years. Only four articles (16%) were published more than 10 years ago. Interestingly, this highlights that this topic has gained momentum in the last 5 years. The types of methodologies that were employed included three systematic reviews (12%), eight retrospective data reviews (32%) and 14 (56%) cohort or prospective studies.

### Ethical considerations

My article followed all ethical standards for research without direct contact with human or animal subjects.

## Results and discussion

The results are discussed according to the objectives of my study and then the themes that were present under each objective.

### Objective one: Description of dysphagia in cervical spinal cord injuries

#### Theme one: The pathophysiology of the swallow in patients with a cervical spinal cord injury

From the 25 articles, 17 articles (68%) discussed the pathophysiology of the swallow and these are described in Table 2.

**TABLE 2:** Types of dysphagia described in the literature.

Types of dysphagia seen in SCI	Cause of the dysphagia and frequency mentioned in the articles (*n*)
Neurological	Cranial nerve injuries (*n* = 7) Neuropathies (*n* = 1) Paralysis (*n* = 2) Transient (*n* = 5)
Structural	Tracheostomy tube *in situ* (*n* = 2) Swelling or stenosis (*n* = 5) Scar tissue formation (*n* = 1)

SCI, spinal cord injury.

By reviewing the description of the dysphagia, it appears that the pharyngeal phase is mostly impacted by a high-level SCI as the most reported symptoms were: residue in pyriform sinus or valleculae, poor laryngeal elevation, poor epiglottic closure, poor base of tongue movement, decreased pharyngeal peristalsis, decreased sensation and impaired upper oesophageal sphincter functioning (Ihalainen 2017; Lee et al. 2016; McRae et al. 2019; Papadopoulou et al. 2013; Shem et al. 2012; Valenzo et al. 2016). According to Wolf and Meiners (2003), patients with a cervical SCI have difficulty protecting their airway because of respiratory difficulties, which is important to identify and manage early.

#### Theme two: Transient nature of the dysphagia in cervical spinal cord injuries

Interestingly, 47% of the articles described the dysphagia as transient in nature within this population. This has implications for the timing of dysphagia screening, assessment and management when considering the development of specific guidelines and practices. If a condition is deemed transient, the need would be to reduce the potential complications in the initial presenting phases. This infers that early identification of dysphagia in this population is required and that screening should be mandatory. This may have an impact on the healthcare team managing these patients because it would be important to agree and collaborate which team members need to conduct the early screening, as SLPs are often not included in the initial management of these patients. It would then become the responsibility of other healthcare team members to be aware of the risk factors of dysphagia and the presentation of the condition. The active engagement of these professions would be important in the early identification process and needs consideration in the planned NHI management procedures.

Given the statements regarding other healthcare team members needing to conduct possible screenings for these patients, it is deemed important to consider each professional’s scope of practice during this development process. It is mentioned in the literature that nursing staff is involved in dysphagia screening, especially in stroke units (Cichero, Heaton & Bassett 2009; Palli et al. 2017). Little to no mention about the role of physiotherapists (PTs) performing the screening of dysphagia is made in the literature and should be considered for further exploration.

### Objective two: Description of the risk factors associated with developing dysphagia in spinal cord injuries

It is important to consider the risk factors that lead to the development of dysphagia in SCI as 21 out of the 25 articles (84%) made a mention of specific risk factors. These risk factors are described in Table 3, which depicts the most common risk factors to the least commonly associated risk factors based on how often they are described in the literature.

**TABLE 3:** Risk factors associated with the development of dysphagia in spinal cord injury.

Factor	Number of articles (*n*)	%
Presence of tracheostomy tube	16	76
Use of ventilation	11	69
Anterior spinal surgery	10	47
Older age	9	42
Brain/neurological injury	9	42
Halo fixation	6	29
Tube feeding	4	19

#### Theme three: Respiratory risk factors

From the information in Table 3, it can be hypothesised that if the respiratory system has been compromised requiring the use of a tracheostomy and/or ventilator, then the possibility of developing dysphagia increases. This is because the breath–swallow cycle has been interrupted and the airway is no longer adequately protected during the swallow, as described above. It is known that patients with a tracheostomy tube are more prone to the development of dysphagia because of the impact of the tube on laryngeal elevation, which has a negative impact on airway closure (Batty 2009). The use of a prolonged ventilation can also lead to post-extubation dysphagia, which is a result of laryngeal trauma because of the presence of the endotracheal tube (Marvin, Thibeault & Ehlenbach 2014). These risk factors coupled with the neurological or structural fallouts from the SCI (Table 2) can further increase the prevalence as well as the severity of presenting dysphagia. When considering the development and implementation of screening protocols and early identification procedures for dysphagia in cervical SCI, it would be important for the initial healthcare professionals to be able to identify the most at-risk patients who need to be prioritised for screening and referred for assessment to SLPs.

#### Theme four: Other risk factors

It is highly plausible that a comorbid traumatic brain injury (TBI) could also result in dysphagia, as dysphagia is reportedly prevalent in 25% – 93% of TBI populations in a developed country context (Lee et al. 2016). In SA, where the mortality rate from trauma incidents is six times higher than the global average (Corrigan, Selassie & Orman 2010), it is pertinent to consider the possible impact of a patient with a TBI and SCI on dysphagia. This requires further investigation.

### Objective three: The impact of dysphagia in patients with a cervical spinal cord injury

#### Theme five: Financial impact

Only 8 (32%) out of the 25 articles referenced the potential impact that a dysphagia has on the patient and on the healthcare facility. Out of these eight articles, only three stated that if a patient with a high SCI also presents with dysphagia, this would lengthen the hospital stay of the patient and the total cost of care (Hayashi et al. 2020; Ihalainen et al. 2017, 2018). This is a potentially important clinical outcome that needs to be considered by all members of the healthcare team. Further consideration is the need to reduce potential medical complications, which have become even more pertinent in the coronavirus disease 2019 (COVID-19) era, where non-emergent medical cases are being discharged at a faster rate (Adams et al. 2020).

#### Theme six: Personal impact of dysphagia on the patient

Not only is dysphagia in SCI, a financial concern, as mentioned above, but the same three articles also mention that dysphagia had a negative impact on the patients, including a decrease in their mood as well as their overall quality of life (Iruthayarajah et al. 2018; Shem et al. 2011, 2012). Quality of life and mood could have a significant impact on the patient’s adherence to rehabilitation. Person-centred care (PCC) is therefore an important framework to consider for all healthcare professionals when rehabilitating these patients (Santana et al. 2018), as the rehabilitation of the respiratory functioning by PTs will also have an impact on the management of dysphagia, thereby possibly making rehabilitation more efficient. Through early identification, complications can be minimised, thereby decreasing hospital stay and increasing quality of life (Iruthayarajah et al. 2018). There is again little research that focusses on PCC in dysphagia as well as for SCI management. This framework could be incorporated into both individual and team practice patterns, but needs further exploration.

### Objective four: Describe the practice patterns in dysphagia management of patients with a spinal cord injury

Only 10 (40%) of the articles mentioned a team approach to managing this patient population.

#### Theme seven: Team members involved in the management of dysphagia in cervical spinal cord injury

Out of the 25 articles included in the review, 10 articles focussed on the management team. The team members who were specifically mentioned included: SLPs, PTs, occupational therapists (OTs), dieticians, nurses and physicians. Given the scope of practice for SCI and dysphagia management, these team members are all appropriate for the management of this population and are all present in most healthcare facilities in SA, both within the public and private sectors.

#### Theme eight: Practice patterns

Of the 10 articles mentioned above that considered the management team, not all mentioned a specific practice model that the healthcare team employed to manage this patient population. The question at hand remains, how do these team members work together in the most efficient manner in a complex healthcare setting such as SA? When considering the documents that are provided by the Southern African Spinal Cord Injury Association (SASCA), the only guidelines available are for mobility and assistive devices (SASCA 2016). There is no mention regarding the presence of dysphagia, and subsequently there is little mention of the team members nor potential management approaches for this population. Given the factors discussed above and the clinical gaps that have emerged, this is an area that requires attention and input from multiple professionals. Once again this will become pertinent not only for policy and guideline development generally, but for the rollout of the NHI and possible undergraduate training.

Anecdotally, in the South African context, a multidisciplinary (MDT) approach is typically followed in all settings. This involves team members working collaboratively in terms of communication and consultation but often independently in terms of scope and practice (Logemann 1994). This model has advantages, but considering the impact of limited resources, the need to identify patients early and the heterogeneous nature of dysphagia within this population, perhaps an alternative model to the MDT approach needs to be explored. Given that there were little data on how teams manage this population, it can be hypothesised that these practice patterns vary internationally. However, from this review, two articles specifically mentioned an interdisciplinary approach (Seidl et al. 2010; Vining et al. 2017), which involves members of different professions often practising across disciplines for patient care (Bewer 2017). The articles in this review highlight the advantages of a transdisciplinary approach, the predominant factor being the efficacy of management teams. Other important factors included structured sessions and good communication. All team members were working towards a common goal to decrease the patients’ length of hospital stay and improve their overall quality of life. There is little research on the interdisciplinary approach in the management of patients with an SCI, yet another identified gap that requires further exploration. Circling back to the point of SA being a resource-restricted setting, an interdisciplinary approach would be a potential solution in a more effective and efficient manner when rehabilitating patients to reduce time with the patients as well as decreasing their overall length of hospital stay. In SA, team members would primarily include physicians, nurses, PTs, dieticians, OTs and SLPs. An interdisciplinary approach would require changes in possible undergraduate teaching curricula as well as practical placements. Each profession has a specific skill set that is highly valued when managing a patient with SCI. Given the need to manage these patients efficiently and not in isolation, it may be important to discuss what knowledge and skills each team member may need to improve current services that are provided. This specifically refers to the need of improving the early identification and screening of at-risk patients.

**Sub-theme one – Practice patterns regarding early identification and screening:** Practice patterns around the early identification of dysphagia are important. It was mentioned as a priority for this population in seven of the articles, and the later management thereof. In SA and other countries, early identification is needed to reduce the long-term impacts of dysphagia. There are only 2643 SLPs registered with the Health Professions Council of South Africa (HPCSA) (Pillay et al. 2020), and not all of those SLPs work in the medical setting, highlighting the limited number of SLPs who are frequently and readily available for screening and early identification of dysphagia. This challenge requires a unique solution. Perhaps the scope of practice documents for physicians, nurses and PTs needs to include the ability to screen and identify at-risk patients for dysphagia, as these team members are responsible for the initial treatment of the patient living with an SCI. The feasibility of this would need to be discussed at individual sites.

**Sub-theme two – Practice patterns regarding management of dysphagia in cervical spinal cord injury:** One of the primary complications of a cervical SCI is problems in the respiratory system, and the management and rehabilitation thereof fall firmly within the scope of the PT (Harvey 2016). Considering the cause of dysphagia, the management of patients living with SCI will require simultaneous respiratory intervention and the SLP cannot and should not treat dysphagia in isolation. This would also require a different and more collaborative team approach. Currently, there is little to no guidance in terms of policies or frameworks in SA for the management of dysphagia in patients with an SCI. This may require PTs to have a broader understanding about dysphagia and for SLPs to have a more detailed input on the respiratory system, which could both be incorporated at an undergraduate level. In terms of how this would play out clinically, more collaboration, research and discussions would be needed. This review merely identified gaps and provided possible suggestions on the way forward.

## Conclusions

Dysphagia is prevalent in patients with a high SCI because of both neurological and structural causes, which impact on the respiratory functioning of the patient. This predominantly leads to symptoms involving the pharyngeal phase, which can lead to aspiration. The untreated dysphagia can lead to various medical complications that will increase the length of hospital stay for the patients and increase their overall cost of care and decrease the patients’ quality of life. By understanding the risk factors that can lead to dysphagia within this population and by shifting practice patterns, screening, early identification and management can be improved in the South African setting. This can be done by potentially utilising other healthcare professionals to screen at-risk patients. Current policies and the NHI management options need to account for the specific management of SCI as well as possible undergraduate curricula for various healthcare professionals. Overall, my review has highlighted gaps and suggestions for both research and clinical considerations. Ultimately, the solutions for a South African context need to account for the contextual complexities and be responsive to the needs of all stakeholders. This could be achieved through greater collaboration between various healthcare professionals.

## Clinical implications

- More epidemiological data are needed on the presence of dysphagia, risk factors for dysphagia and impact of dysphagia in patients with an SCI.
- The roles of various team members in this early identification process need to be explored in interdisciplinary approaches versus a typical MDT approach.
- There is a need to develop context-specific guidelines to assist in the management of dysphagia within this population.
- Considering the unique burden of disease in SA, the risk factors for the development of dysphagia in SCI need to be further investigated. This would ensure that current policies, guidelines and potentially undergraduate training for various healthcare professionals are contextually accurate.
